# Modulation of dielectric properties in low-loss polypropylene-based composites at GHz frequencies: theory and experiment

**DOI:** 10.1038/s41598-022-17173-4

**Published:** 2022-07-30

**Authors:** Konrad Wilczyński, Anna Wróblewska, Agata Daniszewska, Jerzy Krupka, Michał Mrozowski, Mariusz Zdrojek

**Affiliations:** 1 Faculty of Physics, Warsaw University of Technology, 00-662 Warsaw, Poland; 2 Institute of Microelectronics and Optoelectronics, Warsaw University of Technology, 00-662 Warsaw, Poland; 3 Department of Microwave and Antenna Engineering, Faculty of Electronics, Telecommunications and Informatics, Gdańsk University of Technology, 80-233 Gdańsk, Poland

**Keywords:** Materials science, Physics, Engineering, Electrical and electronic engineering

## Abstract

Polymer composites with high dielectric constant and low loss tangent are highly regarded as substrates for modern high-speed electronics. In this work, we analyze the high-frequency dielectric properties of two types of composites based on polypropylene infused with high-dielectric-constant microparticles. Two types of fillers are used: commercial ceramics or titanium oxide (TiO_2_) with different concentrations. The key observation is that adding the fillers causes an increase of dielectric constants by around 100% (for highest loading) up to 4.2 and 3.4, for micro-ceramics and TiO_2_ based composites, respectively. Interestingly, for the TiO_2_ composite, the loss tangent depends on the filler loading volume, whereas the other composite has a slightly increasing tendency, however, being at the level ~ 10^–3^. To explain the experimental results, a theoretical model determined by microwave reflection and transmission through a representative volume element is proposed, which allows the investigation of the impact of volume ratio, grain shape, aggregation, and size on the loss tangent and permittivity evolution. This approach could be used for modeling other low dielectric loss materials with inclusions.

## Introduction

Polymer composites have gained significant attention during the past decade due to their numerous applications in modern life and industry. A vital development sector is the electronic devices working at high frequencies, whose miniaturization is still an unsolved issue due to the requirement to produce new dielectric substrates. The polymer matrix is a promising candidate for substrates because of its flexibility, lightweight, low cost, large-scale production, and high electrical breakdown strength. However, polymer based substrates usually have rather poor dielectric properties in terms of low loss. All the features are crucial for producing new materials with high dielectric constant and low loss tangent that may serve as dielectric substrates for fabricating new RF electronics components, e.g., sensors, antennas, capacitors, and FET transistors. Suitable low-loss materials should exhibit real permittivity > 2 and tangent loss < 10^–2^.

One of the main strategies to increase the dielectric constant in polymer composites is to embed ceramic fillers with a high dielectric constant and intense spontaneous polarization into a polymer matrix. For example, in polyimide composite with TiO_2_ nanoparticles, an increase in the dielectric constant of approximately 10% was observed (for a concentration of 7% TiO_2_) and the loss tangent of the composite has grown approximately four times^1^. The dielectric constant with value 4.4 for the TiO_2_—polydimethylsiloxane compound was measured for a high concentration of inclusion reaching 30 wt%^2^. Implementation of the same concentration of TiO_2_ in PVDF polymer resulted in ${\varepsilon }_{\mathrm{r}}$ value of about 10^3^. In turn, the record value of the dielectric constant (${\varepsilon }_{\mathrm{r}}$ = 133) was obtained for BaTiO3- cyanoethylated cellulose polymer composite for the 51% wt. concentration of barium ceramics^4^. Recently, electrically tunable dielectrics such as Ba_0.6_Sr_0.4_TiO_3_ ceramics have been widely investigated as a filler in polymer. For instance, a PVDF composite with 40% wt. Ba_0.6_Sr_0.4_TiO_3_ reached the value 40 of the dielectric constant^5^. However, this strategy has limitations due to the filler loading threshold. Higher concentrations lead to lower processability and mechanical flexibility^6^. The influence of grain size, cluster formation, or the correlation between different charge parameters and changes in the loss tangent is not yet described in the literature. This information can be obtained by correlating experimental results with theoretical calculations.

The problem of microwave propagation through two-phase composite has been widely studied theoretically in the literature^7^. One of the popular approaches is the use of effective medium theory^8^—a composite is replaced by a medium of isotropic dielectric permittivity, which requires the assumption that the grains in the composite are much smaller than the radiation wavelength. There are many analytical models to calculate the effective composite permittivity—one of the most famous formulas is the Maxwell–Garnett model^9,10^, which assumes the spherical shape of the inclusions and no interaction between them. Multiple extensions of the Maxwell–Garnett model exist, including ellipsoidal shapes and providing better accuracy—a practical overview of the formulas has recently been reported in^11^. However, in the case of composites with a significant permittivity contrast, a large concentration of inclusions, irregular shapes of inclusions, or their clustering, it is not reliable to estimate the effective composite permittivity using only analytical formulas^12–14^. Therefore, a better approach is to perform full-wave electromagnetic simulations. The reported calculations usually consider either a capacitor in the quasi-static regime with a well-defined electric potential^12,15–17^ or microwave propagation through a slab or periodic unit cell of the composite^13,14,18,19^. Multiple methods have been used in the literature to define and extract effective composite permittivity ${\varepsilon }_{\mathrm{eff}}$ with such simulation. The most reliable approach involves calculating the propagation constant $\gamma$ and characteristic impedance $Z$ of the effective medium from the reflection ${S}_{11}$ and transmission ${S}_{21}$ parameters^14,18^, which can be transformed to the equivalent effective permittivity ${\varepsilon }_{\mathrm{eff}}$ and permeability ${\mu }_{\mathrm{eff}}$. Such a procedure does not involve any quasi-static approximations—the calculated effective parameters are strictly related to the scattering and transmission properties of the composite.

The impact of the grain shape on the effective composite permittivity has previously been studied in the literature, but only theoretically and to a limited degree. Reports have concerned ellipsoidal particles with different aspect ratios^20,21^, cylinders^22^, and inclusion shapes containing edges, including two-dimensional polyhedra^15,23,24^, three-dimensional cuboids^12–14,18^, and irregular solid figures^13,14,25^. Some reports also take into account the effect of grain disorder^12,14,24^ (which increases interparticle interactions^26^) or even clustering^13,15^. In each of the listed cases, the real part of the effective permittivity reported is greater than indicated by the well-established Maxwell–Garnett mixing law^9,10^. However, the literature usually assumes that the grain size has no or negligible impact on the permittivity, even though the inclusion dimensions affect their average separation at the fixed volume fraction. In general, the effective medium approximation premise might be invalid if the wavelength is too short.

This work presents a study of the dielectric properties of two types of polypropylene composites infused with micro-ceramics or TiO_2_ nanocrystals. The dielectric constant and a loss tangent of the composites were investigated at 5 GHz or/and 10 GHz. We demonstrate a new approach to simulate the effective permittivity, which provides a better interpretation and generality of the composite microwave properties. The method involves the calculation of S-parameters corresponding to a representative volume element of the composite using a commercially available full-wave simulator, followed by the transformation of the matrix to the four effective permittivities—separate for phase- and impedance-related microwave behaviors in two opposite propagation directions. The approach can be applied to composites with arbitrary inclusions distribution and shapes and provides a natural criterion for the effective medium theory applicability. We comprehensively investigated the impact of volume ratio, grain shape, aggregation, and size on the evolution of the loss tangent and dielectric constant using the proposed simulation method.

## Methods

### Samples and measurement details

In this work, two types of polymer composites were manufactured: polypropylene (PP) filled with micro-ceramics and polypropylene with TiO_2_ nanocrystals. PP was purchased from Resinex in the form of a powder. The TiO_2_ nanocrystals (10 nm diameter) provided by MkNano are also in the form of a powder. The micro-ceramics grains were prepared in a ball mill by grinding a ceramic wafer, purchased from Skyworks, and next the powder was divided into fractions with grain sizes: 20, 25, 32, 56, 60, 90, and 100 um.

The polymer composites of PP with ceramics powder or TiO_2_ were dry-mixed in a lab mixer. We prepared samples with mass fractions in the range of 10–60 wt% for ceramics/PP and 5–40 wt% for TiO_2_/PP composites. Then, the prepared blends were hot-pressed on a hydraulic press with two heating plates heated to 260 °C and pressed with a pressure of approximately 1.7 MPa. To increase accuracy and check repeatability of the results, several samples of each type were prepared.

The samples have thickness in the range from 0.8 to 1.5 mm and lateral dimensions (irregular) of about 40 mm × 40 mm. Measurements of their in-plane permittivity and the dielectric loss tangent have been performed employing a well known split post dielectric resonator technique^27^ at frequencies 5 GHz and 10 GHz. The permittivity and dielectric loss tangent measurement errors, mainly associated with uncertainties in the thickness of samples, were typically in the range of 3–5% because the samples have not been machined after pressing.

For a clear interpretation of experimental data and comparison to theoretical studies, all weight fractions $wt\%$ were converted to the corresponding volume fractions $f$, using the formula^28^:

1 $$\begin{array}{*{20}c} {f = 1/\left( {1 + \frac{{\rho_{i} }}{{\rho_{m} }} \cdot \frac{{1 - {\text{wt}}\% }}{{{\text{wt}}\% }}} \right),} \\ \end{array}$$

where we used the densities ${\rho }_{\mathrm{m}}$ = 0.90 g/cm^3^ of the matrix (polypropylene) and the densities ${\rho }_{\mathrm{i}}$ of the inclusions: 4.17 g/cm^3^ for micro-ceramics and 3.89 g/cm^3^ for TiO_2_ nanocrystals, respectively.

### Computational model motivation and derivation

The most strict and direct method for describing the microwave propagation properties of composites involves calculating the effective propagation constant $\gamma$ and characteristic impedance $Z$ of a representative volume element. The evaluated parameters can be further transformed to the effective microwave material parameters. While the approach is appropriate for the study of composite as a part of microwave devices and waveguides, the most relevant calculation procedure reported so far^14,18^ has two downsides:

- Although the proposed formulas enable evaluating the effective parameters $\gamma$ and $Z$ from the microwave reflection ${S}_{11}$ and transmission parameters ${S}_{21}$, they assume perfect symmetry of the composite representative volume element. In other words, the same reflection (${S}_{11}={S}_{22}$) and transmission (${S}_{21}={S}_{12}$) parameters at both sides of the unit cell have to be either approximated or guaranteed,
- In general, using a single complex parameter—effective permittivity ${\varepsilon }_{\mathrm{eff}}$—can be insufficient to describe microwave propagation through a multi-phase composite. Strictly, the propagation is determined by two complex numbers: $\gamma$ and $Z$. In literature, this issue is resolved by evaluating a possibly non-unit effective permeability ${\mu }_{\mathrm{eff}}$^18^, even if the applied materials are non-magnetic. However, an introduction of fictitious magnetic properties is counter-intuitive and complicates the design of RF devices.

**Figure 1:**
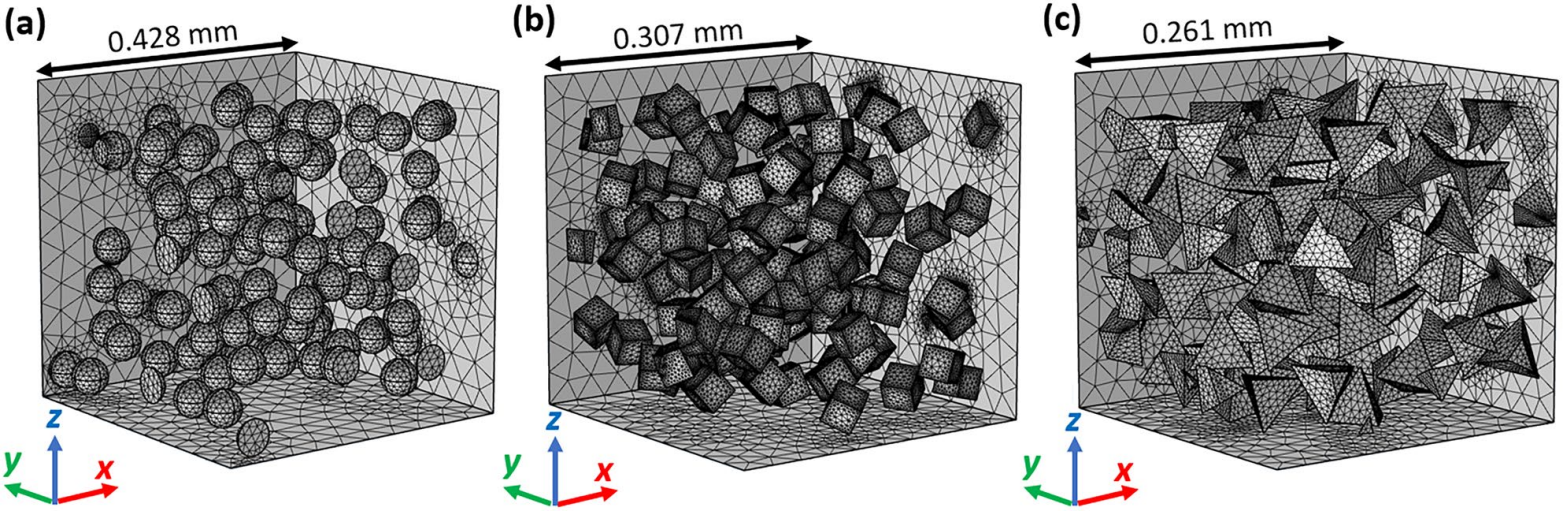
FEM models (with meshes) of the composite with *wt* = 29.6% as implemented in COMSOL Multiphysics 5.4^29^—representative volume elements containing 100 grains (with diameter of 50 µm) in the shape of: (**a**) spheres, (**b**) cubes, (**c**) tetrahedrons.

This paper proposes a simulation model based on the composite’s representative periodic volume element (Fig. 1). The top and bottom boundaries are set to be excitation ports of the TEM wave, and periodic boundary conditions are placed on the sidewalls. The volume element can include an arbitrary number of inclusions, which can intersect the sidewalls but cannot cross the ports—it is assumed that the domain at the ports and behind them is uniform and the same as the composite matrix. Thus, the studied problem is equivalent to plane wave propagation in the composite matrix and its perpendicular incidence onto a virtual, infinite layer, including immersed inclusions. Analysis of microwave transmission through the representative layer enables the study of effective propagation properties of the entire composite, provided that the layer is sufficiently thick to guarantee statistical averaging.

To study the propagation properties of the composite volume element, we evaluate its transmission matrix $\hat{\user2{T}}$, as defined and derived in Supplementary Information:

2 $$\begin{array}{*{20}c} {\hat{\user2{T}} = \left[ {\begin{array}{*{20}c} 1 & 1 \\ {1/Z} & { - 1/Z} \\ \end{array} } \right] \cdot \frac{1}{{S_{12} }}\left[ {\begin{array}{*{20}c} {S_{21} S_{12} - S_{11} S_{22} } & {S_{22} } \\ { - S_{11} } & 1 \\ \end{array} } \right] \cdot \left[ {\begin{array}{*{20}c} 1 & 1 \\ {1/Z} & { - 1/Z} \\ \end{array} } \right]^{ - 1} } \\ \end{array}$$

where $Z=\sqrt{{{\mu }_{0}\mu }_{\mathrm{m}}}/\sqrt{{{\varepsilon }_{0}\varepsilon }_{\mathrm{m}}}$ denotes the characteristic impedance at the layer boundary (where ${\varepsilon }_{m}$, ${\mu }_{m}$—relative permittivity and permeability of the composite matrix, ${\varepsilon }_{0}$, ${\mu }_{0}$—vacuum absolute permittivity and permeability) and ${S}_{11}$, ${S}_{12}$, ${S}_{21}$, ${S}_{22}$ are the S-parameters of the volume element. As the two eigenvalues of the $\hat{\user2{T}}$ matrix are not degenerate, the matrix is diagonalizable and can be written in the following general form:

3 $$\begin{array}{*{20}c} {\hat{\user2{T}} = \left[ {\begin{array}{*{20}c} 1 & 1 \\ {1/Z_{{{\text{eff}}}}^{\left( + \right)} } & { - 1/Z_{{{\text{eff}}}}^{\left( - \right)} } \\ \end{array} } \right] \cdot \left[ {\begin{array}{*{20}c} {e^{{ - \gamma_{{{\text{eff}}}}^{\left( + \right)} d}} } & 0 \\ 0 & {e^{{ + \gamma_{{{\text{eff}}}}^{\left( - \right)} d}} } \\ \end{array} } \right] \cdot \left[ {\begin{array}{*{20}c} 1 & 1 \\ {1/Z_{{{\text{eff}}}}^{\left( + \right)} } & { - 1/Z_{{{\text{eff}}}}^{\left( - \right)} } \\ \end{array} } \right]^{ - 1} ,} \\ \end{array}$$

where we set $d$ equal to the thickness of the layer. Matrix $\hat{\user2{T}}$ in Eq. (3) can be interpreted as the transmission matrix of an effective medium with separate characteristic impedances ${Z}_{\mathrm{eff}}^{(+)}$, ${Z}_{\mathrm{eff}}^{(-)}$ and propagation constants ${\gamma }_{\mathrm{eff}}^{(+)}$, ${\gamma }_{\mathrm{eff}}^{(-)}$ for two propagation directions. In general, the parameters indexed with “ + ” and “−” might be different—if the representative layer is not symmetrical. The above characteristic impedances and propagation constants could now be transformed to the effective permittivity and permeability of the representative composite layer. However, for more straightforward interpretation and application, it is beneficial to set ${\mu }_{\mathrm{eff}}=1$, and define separate effective permittivities corresponding to the $\gamma_{{{\text{eff}}}}^{\left( + \right)}$, $\gamma_{{{\text{eff}}}}^{\left( - \right)}$ (phase-related) and $Z_{{{\text{eff}}}}^{\left( + \right)}$, $Z_{{{\text{eff}}}}^{\left( - \right)}$ (impedance-related) parameters:

4 $$\begin{array}{*{20}c} {\varepsilon_{{{\text{eff}}}}^{\gamma + } = \left( {\frac{{\gamma_{{{\text{eff}}}}^{\left( + \right)} }}{{jk_{0} }}} \right)^{2} , \varepsilon_{{{\text{eff}}}}^{\gamma - } = \left( {\frac{{\gamma_{{{\text{eff}}}}^{\left( - \right)} }}{{jk_{0} }}} \right)^{2} , \varepsilon_{{{\text{eff}}}}^{Z + } = \left( {\frac{{\sqrt {\mu_{0} /\varepsilon_{0} } }}{{Z_{{{\text{eff}}}}^{\left( + \right)} }}} \right)^{2} , \varepsilon_{{{\text{eff}}}}^{Z - } = \left( {\frac{{\sqrt {\mu_{0} /\varepsilon_{0} } }}{{Z_{{{\text{eff}}}}^{\left( - \right)} }}} \right)^{2} ,} \\ \end{array}$$

where ${k}_{0}=2\pi /{\lambda }_{0}$ is the phase constant in vacuum. Note that in the case of a uniform medium, all the above four effective permittivities are equal. The four permittivities (Eq. 4) can be evaluated for an arbitrary composite, provided that the impact of interference effects on power transmission is negligible (most of the power is transmitted with TEM waves). If the four permittivities are similar, this indicates the applicability of the effective medium theory for a specific composite. If the phase-related ${\varepsilon }_{\mathrm{eff}}^{\gamma +}$, ${\varepsilon }_{\mathrm{eff}}^{\gamma -}$ and impedance-related ${\varepsilon }_{\mathrm{eff}}^{Z+}$, ${\varepsilon }_{\mathrm{eff}}^{Z-}$ permittivities were significantly different, and separate effective dielectric constants would have to be used to match impedances between two media (or waveguides) and on the purpose of attenuation or phase change study.

Two aspects should be taken into consideration when evaluating the effective dielectric constants (Eq. 4) of the composite. First, the effective permittivity of a representative finite-sized volume element depends on the random distribution of inclusions and, additionally, on the microwave polarization direction, resulting in an anisotropic dielectric constant. The smaller the considered supercell, the more significant impact of cell periodicity and anisotropy on the results^16^. However, the effective isotropic permittivity can be evaluated as the mean value over multiple random structures and all components of the anisotropic permittivity; this approach leads to the averaging of all statistical fluctuations^12,15,16,30^. Secondly, we recorded that the impedance-related permittivities ${\varepsilon }_{\mathrm{eff}}^{Z+}$ and ${\varepsilon }_{\mathrm{eff}}^{Z-}$ can depend on the direction (sign) of microwave propagation due to supercell asymmetry. In the case of low-loss composites, the difference between ${\varepsilon }_{\mathrm{eff}}^{Z+}$ and ${\varepsilon }_{\mathrm{eff}}^{Z-}$ is primarily in the imaginary part. However, we did not observe any noticeable difference between ${\varepsilon }_{\mathrm{eff}}^{\gamma +}$ and ${\varepsilon }_{\mathrm{eff}}^{\gamma -}$ in our studies. Additionally, we checked that if the permittivities ${\varepsilon }_{\mathrm{eff}}^{Z+}$ and ${\varepsilon }_{\mathrm{eff}}^{Z-}$ corresponding to both propagation directions are averaged, one obtains the correct permittivity value—very close to ${\varepsilon }_{\mathrm{eff}}^{\gamma +}$ and ${\varepsilon }_{\mathrm{eff}}^{\gamma -}$ in both real and imaginary parts. Therefore, to study composite's effective permittivity with a given inclusions concentration, we generated five pseudo-random supercells and evaluated each of the four permittivities (Eq. 4) for the two perpendicular axes: *x* and *y* (parallel to the cube walls). Phase-related ${\varepsilon }_{\mathrm{eff}}^{\gamma }$ and impedance-related ${\varepsilon }_{\mathrm{eff}}^{Z}$ permittivities were evaluated as the averages over 20 individual values: for two propagation directions (signs “+” and “−”), for two axes (*x* and *y*), and for each of the five supercells:

5 $$\begin{array}{*{20}c} {\varepsilon_{{{\text{eff}}}}^{\gamma } = \frac{1}{4N}\mathop \sum \limits_{n = 1}^{N} \left( {\varepsilon_{{\begin{array}{*{20}c} {\text{eff}, x} \\ n \\ \end{array} }}^{\gamma + } + \varepsilon_{{\begin{array}{*{20}c} {\text{eff}, y} \\ n \\ \end{array} }}^{\gamma + } + \varepsilon_{{\begin{array}{*{20}c} {\text{eff}, x} \\ n \\ \end{array} }}^{\gamma - } + \varepsilon_{{\begin{array}{*{20}c} {\text{eff}, y} \\ n \\ \end{array} }}^{\gamma - } } \right), \varepsilon_{{{\text{eff}}}}^{Z} = \frac{1}{4N}\mathop \sum \limits_{n = 1}^{N} \left( {\varepsilon_{{\begin{array}{*{20}c} {\text{eff}, x} \\ n \\ \end{array} }}^{Z + } + \varepsilon_{{\begin{array}{*{20}c} {\text{eff}, y} \\ n \\ \end{array} }}^{Z + } + \varepsilon_{{\begin{array}{*{20}c} {\text{eff}, x} \\ n \\ \end{array} }}^{Z - } + \varepsilon_{{\begin{array}{*{20}c} {\text{eff}, y} \\ n \\ \end{array} }}^{Z - } } \right), } \\ \end{array}$$

where $n$ denotes supercells and $N$ = 5 is the number of supercells.

We implemented all computational models in the COMSOL Multiphysics 5.4 software^29^, based on the finite-element method (FEM)—the electromagnetic field vectors were evaluated on a discrete set of points in the 3D space. The solution between the points was interpolated using quadratic order basis functions. In the models, the maximal distance between the mesh points was equal to 0.1 of the supercell side length, which was much lower than the microwave wavelength at 5 GHz (60 mm in vacuum). In the case of inclusions with edges, we used a dense mesh at each edge with a maximum point separation equal to 0.1 of the edge length, and the growth rate of the mesh element was equal to 1.5. This provided a proper description of the electromagnetic field localized on the edges and corners of the inclusions.

## Results and discussion

### Ceramics/polypropylene composite

#### Effect of the filler fraction

**Figure 2:**
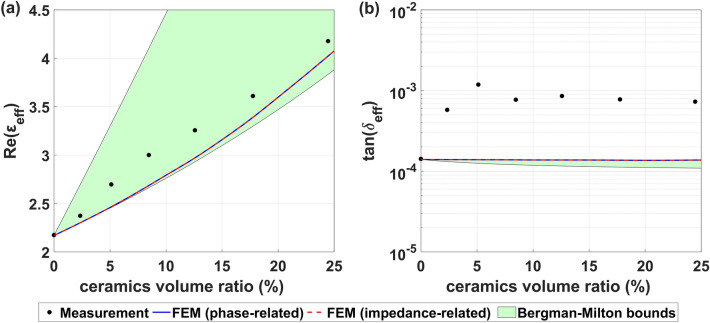
Correlation of the ceramic concentration on the composite (**a**) real dielectric constant and (**b**) loss tangent at the frequency of 5 GHz. Measurement, simulation results, and analytical Bergman-Milton bounds are presented.

The results of the dielectric constant and loss tangent measurements for PP-filled commercial micro-ceramics are shown in Fig. 2 (see dots). We observed that the dielectric constant increases with the ceramics concentration and reaches 4.2 for the maximal filler concentration. In the case of the loss tangent, we recorded a significant increase to about ~ 10^–3^ at the filler volume fraction of 5%; however, no further evolution was observed at greater filler concentrations. The physical origin of the observed increase in real permittivity can be explained in terms of the well-known polarization mechanism^10^. Suppose the composite is located in an external electric field. In this case, dipole moments are induced within each inclusion, which can be interpreted as the induction of effective charges on the grains’ surfaces. As a result, the electric field distribution in the composite is affected, which is seen by the microwave as a medium with new effective permittivity, provided that the wavelength is sufficiently large. The alternating electric field can also interact with current carriers leading to Joule heating. Additionally, energy dissipation due to non-resonant interaction between electric field and phonons can occur—within three-quantum, four-quantum, and quasi-Debye loss mechanisms^31^. To quantitatively understand the evolution of measured dielectric parameters, we performed a study using a new simulation method, which enabled us to include the effects of the fillers’ geometrical structure, such as the formation of agglomerates in the prepared composite and grain size.

The computational permittivity of the composite was evaluated separately on the grounds of the effective propagation constants (“phase-related”) and the grounds of the effective impedance (“impedance-related”)—as defined in Eq. (5). Each of the studied supercells contained *N* = 100 spherical grains with diameter *d* = 56 µm (Fig. 1a), and the corresponding side length *a* of the supercell was evaluated using the equation:

6 $$\begin{array}{*{20}c} {f \cdot a^{3} = N \cdot V_{{\text{i}}} ,} \\ \end{array}$$

where $f$ stands for the volume fraction of the inclusions and ${V}_{\mathrm{i}}=4\pi /3\cdot {\left(d/2\right)}^{3}$ is the volume of a single inclusion. We used isotropic dielectric parameters $\varepsilon_{{\text{m}}}^\prime$ = 2.17, ${\text{tan }} \delta_{{\text{m}}}$= 1.4 × 10^–4^ for the matrix and $\varepsilon_{{\text{i}}}^{\prime}$ = 34.5, ${\text{tan }} \delta_{{\text{i}}}$= 1.0 × 10^–4^ for the inclusions. The calculated sets of permittivities ${\varepsilon }_{\mathrm{eff}}^{\gamma }$ and ${\varepsilon }_{\mathrm{eff}}^{Z}$ for grains volume fraction $f$ are presented in Fig. 2. The convergence of the simulation results using *N* = 100 inclusions and the insignificance of the statistical fluctuations caused by random grain distributions were confirmed in Supplementary Information. The values $\varepsilon_{{{\text{eff}}}}^{\gamma }$ and $\varepsilon_{{{\text{eff}}}}^{Z}$ perfectly agree with each other, which indicates that the composite can be treated as an effective medium. Additionally, theoretical Bergman-Milton bounds^32^ are presented for comparison (green area). They indicate the range of possible real permittivities $\varepsilon_{{{\text{eff}}}}^\prime$ and loss tangents ${\text{tan }} \delta_{{{\text{eff}}}}$ in a macroscopically isotropic composite with fixed values of $\varepsilon_{{\text{m}}}^\prime$, ${\text{tan }} \delta_{{\text{m}}}$, $\varepsilon_{{\text{i}}}^\prime$, ${\text{tan }} \delta_{{\text{i}}}$, and $f$.

The real measured permittivities are slightly greater than the simulated ones, indicating different properties of the samples not present in the model—probably a non-spherical shape of the inclusions or their agglomeration. In the case of real permittivities $\varepsilon_{{{\text{eff}}}}^\prime$, the measured values are inside theoretical Bergman–Milton bounds for each fraction of inclusions. Therefore, a grain shape exists, which would guarantee an agreement between the experimental and simulated values of $\varepsilon_{{{\text{eff}}}}^\prime$. The experimental loss tangents are greater than the simulated ones by almost an order of magnitude for any non-zero concentration of inclusions. As this result violates the Bergman-Milton bounds, effective medium theory cannot explain the high increase of the measured loss tangent. One of the possible origins of the additional loss can be a scattering of microwaves on the grains and a power leakage perpendicularly to the wave propagation direction. This effect is not taken into account in the simulation model, as it considers an infinitely wide composite layer. On the other hand, such loss increase has also been reported in the case of the PE/SCT composite at 8 GHz and supposed to be due to interaction of the alternating electric field with phonons or extrinsic factors like defects, interfaces, shape and size of the grains, and micropores^33^. A manifestation of increased loss has recently also been reported for polymer composites measured at terahertz frequencies and attributed to the impact of reduced crystallinity, the presence of free carriers, and improper sample pressing^34^.

#### Effect of grain size, shape, and aggregation

**Figure 3:**
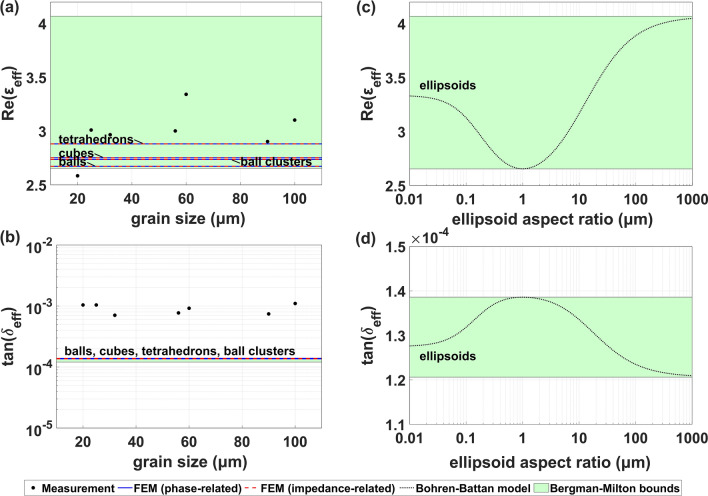
Impact of the grain size, shape, and clustering on (**a**,**c**) real part, and (**b**,**d**) loss tangent of the composite dielectric constant with *wt* = 29.6% at the frequency of 5 GHz. Results for non-ellipsoidal (**a**,**b**) and ellipsoidal (**c**,**d**) inclusion shapes are presented. Theoretical Bergman-Milton bounds are presented for comparison.

Here, we perform a comprehensive study of the effect of grain shape, clustering, and dimensions of inclusions in the ceramics/polypropylene composite. We considered pseudorandom composites with different inclusion diameters $d$ (maximal distance between two points)—in the range 20–100 µm—with the computational model and experimentally. Additionally, we considered different inclusion shapes in the simulation: balls (Fig. 1a), cubes (Fig. 1b), and tetrahedra (Fig. 1c), whose grain volumes ${V}_{\mathrm{i}}$ equal $\pi /6 \cdot d^{3}$, $\sqrt 3 /9 \cdot d^{3}$, and $\sqrt 2 /12 \cdot d^{3}$, respectively. The proper unit cell side length was calculated in each case using Eq. (6) for the relevant inclusion volume ${V}_{\mathrm{i}}$, guaranteeing the desired volume fraction $f$—corresponding to *wt* = 29.6% in Eq. (1). In the case of the experiment, only the maximal grain diameter is controllable, while the actual shapes of the inclusions depend on the preparation method. The study was carried out at a frequency of 5 GHz. As presented in Fig. 3a,b, the computational results indicate that the grain diameter, regardless of the inclusion shape, has a negligible impact on the effective dielectric constant: the wavelength is sufficiently large compared to the grain dimensions. However, we observe significant fluctuations of the real part of the experimental permittivities, which have to be attributed to different effects.

The results presented in Fig. 3a,b indicate a slight dependence of the effective composite permittivity on the inclusion shape. The more irregular the grain (the smaller volume for a given diameter *d*), the more edges and corners the electromagnetic field localizes on^14,25^, and the greater the real permittivity. We also considered an effect of grain aggregation in the case of the spherical inclusions—by forcing the inclusions to form four-element, tetrahedron-shaped clusters. The real permittivity increase obtained for the ball aggregates is similar to that obtained with cubic grains, which is an effect of electromagnetic field localization in clusters' edges and corners. However, the observed changes are much below the fluctuations of the experimental results. The fluctuations are still inside the Bergman-Milton bounds.

To further study the range of the possible effective composite permittivities, we considered ellipsoidal inclusions with different aspect ratios (ratios of their semi-axes). The ground of the analysis is the generalized Maxwell–Garnett formula (reported by Bohren and Battan) for randomly oriented inclusions^35,36^:

7 $$\begin{array}{*{20}c} {\varepsilon_{{{\text{eff}}}} = \varepsilon_{{\text{m}}} \times \left( {1 + \frac{{\frac{f}{3}\mathop \sum \nolimits_{j = x,y,z} \frac{{\varepsilon_{{\text{i}}} - \varepsilon_{{\text{m}}} }}{{\varepsilon_{{\text{m}}} + N_{j} \left( {\varepsilon_{{\text{i}}} - \varepsilon_{{\text{m}}} } \right)}}}}{{1 - \frac{f}{3}\mathop \sum \nolimits_{j = x,y,z} \frac{{N_{j} \left( {\varepsilon_{{\text{i}}} - \varepsilon_{{\text{m}}} } \right)}}{{\varepsilon_{{\text{m}}} + N_{j} \left( {\varepsilon_{{\text{i}}} - \varepsilon_{{\text{m}}} } \right)}}}}} \right) ,} \\ \end{array}$$

where ${N}_{j}$ stands for the depolarization factor:

8 $$\begin{array}{*{20}c} {N_{j} = \frac{{a_{x} a_{y} a_{z} }}{2}\mathop \int \limits_{0}^{\infty } \frac{ds}{{\left( {s + a_{j}^{2} } \right)\sqrt {\left( {s + a_{x}^{2} } \right)\left( {s + a_{y}^{2} } \right)\left( {s + a_{z}^{2} } \right)} }}, j = x,y,z,} \\ \end{array}$$

and ${a}_{x}$, ${a}_{y}$, ${a}_{z}$ are the ellipsoid’s semi-axes. The inclusion dimensions do not appear in the formulas. Although the model (Eq. 7) does not take into account interaction between the inclusions, the results in Fig. 3c,d indicate that ellipsoidal grains with aspect ratio much above 1 (oblate) or much below 1 (prolate) involve a significant increase of the real effective permittivity—the increase can be much greater than due to clustering or the presence of edges. This suggests that the reason for the high fluctuations observed in the experiment might be the aspect ratio randomness of the sieved grains—even if their maximal diameter (doubled semi-major axis of the ellipsoid) is fixed.

### TiO_2_/polypropylene composite

**Figure 4:**
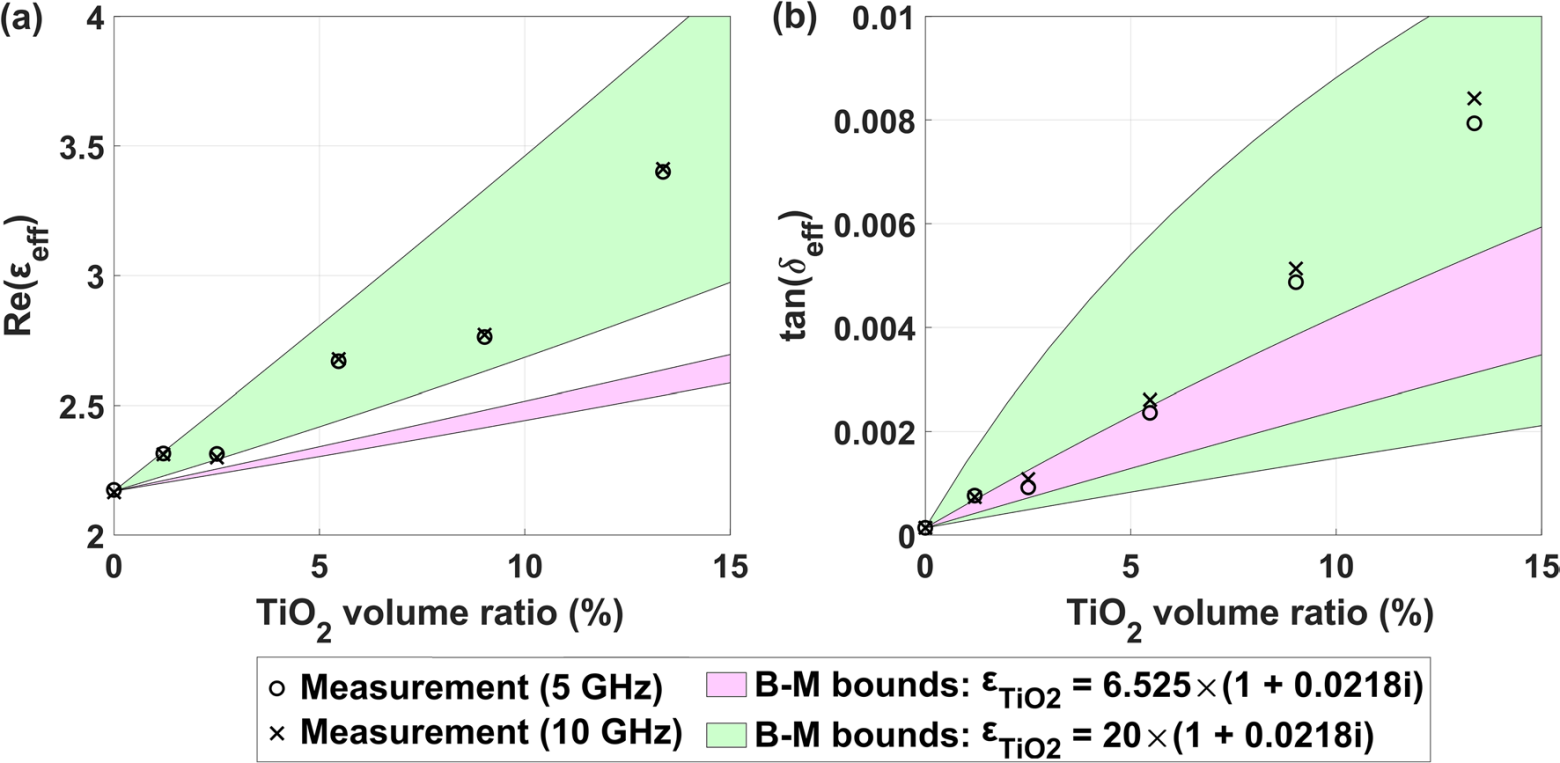
Correlation of the concentration of TiO_2_ nanocrystals on the composite (**a**) real dielectric constant and (**b**) loss tangent at frequencies of 5 GHz and 10 GHz. For comparison, theoretical Bergman-Milton bounds are presented for two complex permittivities of TiO_2_.

Figure 4 presents the measured results of the complex dielectric function $\varepsilon_{{{\text{eff}}}} = \varepsilon_{{{\text{eff}}}}^\prime - j\varepsilon_{{{\text{eff}}}}^{\prime\prime}$ of the TiO_2_/polypropylene composite at 5 GHz and 10 GHz frequencies. We recorded an increasing correlation for $\varepsilon_{{{\text{eff}}}}^\prime$ and ${\text{tan }} \delta_{{{\text{eff}}}}$ as a function of containing TiO_2_ in polymer matrix. However, we observed no noticeable difference between the datasets for different frequencies, suggesting that the effect of permittivity dispersion in this microwave range is negligible. Moreover, as the loss tangent does not change between frequencies 5 GHz and 10 GHz, the source of the microwave loss is probably not Joule heating (as the imaginary part of the dielectric constant $\varepsilon^{\prime\prime}$ related to electrical conductivity is proportional to $\sigma /f$). Therefore, the most probable reason for the observed microwave loss in TiO_2_-based composites is the interaction between electric field and phonons^31^. The physical origin of the observed increasing composite’s real permittivity $\varepsilon_{{{\text{eff}}}}^\prime$ is similar to ceramics/polypropylene composite and involves the polarization mechanism.

In order to validate the experimental results using theoretical studies, it is necessary to know the dielectric function of the TiO_2_ nanograins with anatase structure in the gigahertz frequency range. However, the choice of the dielectric function of TiO_2_ nanograins is ambiguous. The reported experimental values are $\varepsilon_{{{\text{TiO}}2}}^\prime$ = 6.525 and ${\text{tan }} \delta_{{{\text{TiO}}2}}$ = 0.0218 for TiO_2_ in pellet form (with possible air traps) at 2.45 GHz with a particle size of 369 nm^37^. In the frequency range from 1 to 18 GHz for nanocrystals with a diameter of 15 nm, the values of $\varepsilon_{{{\text{TiO}}2}}^\prime$ = 6.1–6.5 and ${\text{tan }} \delta_{{{\text{TiO}}2}}$ ≤ 0.05 are reported^38^. However, the possible values of the effective dielectric constant of the composite, as limited by the theoretical Bergman–Milton bounds^32^ applied for the former reported TiO_2_ dielectric function (pink area), are clearly below the measurement results for both $\varepsilon_{{{\text{eff}}}}^\prime$ and ${\text{tan }} \delta_{{{\text{eff}}}}$, indicating the underestimated real permittivity $\varepsilon_{{{\text{TiO}}2}}^\prime$ of the TiO_2_ nanograins. Interestingly, the theoretically allowed values of $\varepsilon_{{{\text{eff}}}}$ agree well with the experimental data for $\varepsilon_{{{\text{TiO}}2}}^\prime$ increased to about 20 (green area), with the same value of ${\text{tan }} \delta_{{{\text{TiO}}2}}$. While the reported values of $\varepsilon_{{{\text{TiO}}2}}^\prime$ for TiO_2_ anatase nanograins, as measured in the gigahertz regime, are clearly underestimated, there exist reliable presumptions to consider a greater effective $\varepsilon_{{{\text{TiO}}2}}^\prime$ for the TiO_2_ nanograins in the composite. Firstly, although the permittivity $\varepsilon_{{{\text{TiO}}2}}^\prime \sim$ 6 agrees well with the reported bulk electronic permittivities ${\varepsilon }_{\mathrm{TiO}2}^{\infty }$ of anatase TiO_2_, as obtained with both measurements^39,40^ and first-principles simulations^41,42^, this value is applicable above the phonon frequencies. In contrast, the static value ${\varepsilon }_{\mathrm{TiO}2}^{0}$ is more appropriate below this regime. The reported values of ${\varepsilon }_{\mathrm{TiO}2}^{0}$ are anisotropic, and of the order of 45 and 23 for polarizations perpendicular and parallel to the c axis, respectively^39,42^. Although the permittivity tends to decrease significantly for sub-micron thick films^43–45^, the mechanism of the polarizability reduction might be complex and result in different behavior for nanograins in the composite. Moreover, an increase of $\varepsilon_{{{\text{TiO}}2}}^\prime$ in TiO_2_ anatase nanograins upon hydrogenation by about 100% was reported^38^—a similar effect might be present in nanograins surrounded by polypropylene. Further study is needed to provide the dielectric function of TiO_2_ filler appropriate for modeling the composites—potentially affected by the nano-meter grains’ dimensions and the presence of the polypropylene matrix.

Similar to the studies presented for ceramics/polypropylene composites, we simulated the effects of grain size, shape, and aggregation on dielectric properties of TiO_2_/polypropylene composites, using the isotropic dielectric parameters of all TiO_2_ nanocrystals equal $\varepsilon_{{{\text{TiO}}2}}^\prime$ = 20 and ${\text{tan }} \delta_{{{\text{TiO}}2}}$ = 0.0218 (see the above discussion). The calculated results, as presented in Supplementary Information, indicate no noticeable impact of the grain size on the effective dielectric constant of the composites. We also showed that both real permittivity and loss tangent are affected by the shape and clustering of the inclusions—the more irregular the grain shape, the greater the dielectric parameters.

## Conclusions

We performed a comprehensive study of real permittivity $\varepsilon_{{{\text{eff}}}}^\prime$ and loss tangent ${\text{tan }} \delta_{{{\text{eff}}}}$ of two representative low-loss composites based on polypropylene (PP) and high-dielectric-constant filler microparticles—micro-ceramics and titanium oxide (TiO_2_)—with various concentrations, at GHz frequency. An increasing correlation for $\varepsilon_{{{\text{eff}}}}^\prime$ as a function of the filler fraction was observed for both types of fillers. For the maximal considered grain concentrations, this enabled to obtain the real permittivities of 4.2 and 3.4 in ceramics/PP and TiO_2_/PP composites, respectively. In the case of ceramics-PP composite, we confirmed theoretically that the presence of fillers impacts the effective propagation constant and characteristic impedance of the composite, both of which can be described in terms of the same value of the effective dielectric constant in the gigahertz regime. Additionally, we calculated that the real permittivity is significantly affected by the aspect ratio of ellipsoid-shaped inclusions, while the impacts of grain aggregation, presence of edges, and size are much less significant.

In the case of loss tangent, we observed different behaviors for the two types of fillers. Loss tangent in TiO_2_/PP composite increases with nanograins concentration which is clearly the effect of microwave loss of the filler. Contrary, loss tangent in ceramics/PP composite has an increasing tendency below the filler volume fraction of 5% and remains constant and equal to about 10^–3^ above this fraction. We attributed the intriguing observation to scattering of microwaves on the grains and a power leakage perpendicularly to the wave propagation direction in the composite. The increased loss can also result from an evolved microstructure of the fillers and/or matrix in the composite and interface effects.

## Supplementary Information

Supplementary Information.

## Data Availability

The data that support the findings of this study are available from the corresponding author upon reasonable request.

## Code availability

All non-commercial numerical codes to reproduce the findings of this study are available from the corresponding author upon reasonable request.

## References

[1] Feng Y (2013). Effect of nano-TiO2 on the polarization process of polyimide/TiO2 composites. Mater. Lett..

[2] Ouyang G, Wang K, Chen XY (2012). TiO2 nanoparticles modified polydimethylsiloxane with fast response time and increased dielectric constant. J. Micromech. Microeng..

[3] Gan WC, Majid WHA (2014). Effect of nano-TiO2 on enhanced pyroelectric activity of PVDF composite. Smart Mater. Struct..

[4] Chiang CK, Popielarz R (2002). Polymer composites with high dielectric constant. Ferroelectrics.

[5] Hu, G. *et al.* Preparation and dielectric properties of poly(vinylidene fluoride)/Ba0.6Sr0.4TiO3 composites. *J. Alloys Compounds* **619**, 686–692 (2015).

[6] Zhang X (2014). Ultrahigh energy density of polymer nanocomposites containing BaTiO3@TiO2 nanofibers by atomic-scale interface engineering. Adv. Mater..

[7] Brosseau C (2006). Modelling and simulation of dielectric heterostructures: A physical survey from an historical perspective. J. Phys. D: Appl. Phys..

[8] Choy, T. C. *Effective Medium Theory: Principles and Applications*. (Oxford University Press, 2015).

[9] Garnett, J. C. M. & Larmor, J. Colours in metal glasses and in metallic films. *Philos. Trans. R. Soc. Lond. Ser. A, Containing Papers of a Mathematical or Physical Character* **203**, 385–420 (1904).

[10] Markel, V. A. Introduction to the Maxwell Garnett approximation: tutorial. *J. Opt. Soc. Am. A, JOSAA* **33**, 1244–1256 (2016). 10.1364/JOSAA.33.001244 27409680

[11] Yao H-Y, Lin Y-W, Chang T-H (2021). Dielectric properties of BaTiO3–epoxy nanocomposites in the microwave regime. Polymers.

[12] Cheng Y (2008). Modeling and simulation for effective permittivity of two-phase disordered composites. J. Appl. Phys..

[13] Wu D, Chen J, Liu C (2007). Numerical evaluation of effective dielectric properties of three-dimensional composite materials with arbitrary inclusions using a finite-difference time-domain method. J. Appl. Phys..

[14] Pickles AJ, Kilgore IM, Steer MB (2013). Electromagnetic properties of disordered three-dimensional mixtures. IEEE Access.

[15] Sareni B, Krähenbühl L, Beroual A, Brosseau C (1997). Effective dielectric constant of random composite materials. J. Appl. Phys..

[16] Jylha L, Sihvola AH (2005). Numerical modeling of disordered mixture using pseudorandom simulations. IEEE Trans. Geosci. Remote Sens..

[17] Qasim SA, Gupta N (2021). Computation of effective properties of graded and layered dielectrics. IEEE Trans. Dielectr. Electr. Insul..

[18] Wu WM (2014). Studies of permittivity and permeability of dielectric matrix with cuboid metallic inclusions in different orientations. J. Adv. Dielect..

[19] Choroszucho A, Butrylo B, Steckiewicz A, Stankiewicz JM (2020). Determination of the effective electromagnetic parameters of complex building materials for numerical analysis of wireless transmission networks. Electronics.

[20] Polder D, van Santeen JH (1946). The effective permeability of mixtures of solids. Physica.

[21] Shu-Ang Z (1991). A material multipole theory of elastic dielectric composites. Int. J. Solids Struct..

[22] Sareni B, Krähenbühl L, Beroual A, Brosseau C (1996). Effective dielectric constant of periodic composite materials. J. Appl. Phys..

[23] Mejdoubi A, Brosseau C (2006). Finite-element simulation of the depolarization factor of arbitrarily shaped inclusions. Phys. Rev. E.

[24] Cheng H, Torquato S (1997). Electric-field fluctuations in random dielectric composites. Phys. Rev. B.

[25] Pickles AJ, Steer MB (2013). Effective permittivity of 3-D periodic composites with regular and irregular inclusions. IEEE Access.

[26] Lian H, Qin J, Freed KF (2018). Dielectric virial expansion of polarizable dipolar spheres. J. Chem. Phys..

[27] Krupka J (2001). Uncertainty of complex permittivity measurements by split-post dielectric resonator technique. J. Eur. Ceram. Soc..

[28] Xia X, Wang Y, Zhong Z, Weng GJ (2016). A theory of electrical conductivity, dielectric constant, and electromagnetic interference shielding for lightweight graphene composite foams. J. Appl. Phys..

[29] *COMSOL 5.4*. (COMSOL AB, 2018).

[30] Chen, X., Cheng, Y., Wu, K. & Wu, S. Interfacial polarization and its influence on effective complex permittivity of mixtures. in *2008 International Symposium on Electrical Insulating Materials (ISEIM 2008)* 238–241 (2008).

[31] Gurevich VL, Tagantsev AK (1991). Intrinsic dielectric loss in crystals. Adv. Phys..

[32] Milton GW (1981). Bounds on the complex permittivity of a two-component composite material. J. Appl. Phys..

[33] Subodh G, Deepu V, Mohanan P, Sebastian MT (2009). Dielectric response of high permittivity polymer ceramic composite with low loss tangent. Appl. Phys. Lett..

[34] Bergen MH, Mitchell ME, Mellors EM, Holzman JF (2021). Manifestations of loss in terahertz polymer composites. Opt. Mater. Express.

[35] Bohren CF, Battan LJ (1982). Radar backscattering of microwaves by spongy ice spheres. J. Atmos. Sci..

[36] Sihvola A (2000). Mixing rules with complex dielectric coefficients. Subsurf. Sens. Technol. Appl..

[37] Horikoshi, S. *et al.* Microwave-specific effects in various TiO2 specimens. Dielectric properties and degradation of 4-chlorophenol. *J. Phys. Chem. C* **113**, 5649–5657 (2009).

[38] Xia T, Zhang C, Oyler NA, Chen X (2014). Enhancing microwave absorption of TiO2 nanocrystals via hydrogenation. J. Mater. Res..

[39] Gonzalez RJ, Zallen R, Berger H (1997). Infrared reflectivity and lattice fundamentals in anatase TiO2. Phys. Rev. B.

[40] Wemple SH (1977). Optical oscillator strengths and excitation energies in solids, liquids, and molecules. J. Chem. Phys..

[41] Asahi R, Taga Y, Mannstadt W, Freeman AJ (2000). Electronic and optical properties of anatase TiO2. Phys. Rev. B.

[42] Mikami M, Nakamura S, Kitao O, Arakawa H (2002). Lattice dynamics and dielectric properties of TiO2 anatase: A first-principles study. Phys. Rev. B.

[43] Takeuchi M, Itoh T, Nagasaka H (1978). Dielectric properties of sputtered TiO2 films. Thin Solid Films.

[44] Rausch N, Burte EP (1993). Thin TiO2 films prepared by low pressure chemical vapor deposition. J. Electrochem. Soc..

[45] Zhou L, Hoffmann RC, Zhao Z, Bill J, Aldinger F (2008). Chemical bath deposition of thin TiO2-anatase films for dielectric applications. Thin Solid Films.

