# Supplementary material for: Modulation of dielectric properties in low-loss polypropylene-based composites at GHz frequencies: theory and experiment
Source: Sci Rep. 2022 Jul 30;12:13104. doi: 10.1038/s41598-022-17173-4 (PMC9338949; doi:10.1038/s41598-022-17173-4)
Supplement: Supplementary file 1 — Supplementary Information. [file 41598_2022_17173_MOESM1_ESM.pdf]

Modulation of dielectric properties in low-loss polypropylene-based composites at GHz frequencies – theory and experiment

K. Wilczyński*[1], A. Wróblewska[1], A. Daniszewska[1], J. Krupka[2], M. Mrozowski[3], M. Zdrojek[1]

[1] Faculty of Physics, Warsaw University of Technology, 00-662 Warsaw, Poland

[2] Institute of Microelectronics and Optoelectronics, Warsaw University of Technology, 00-662 Warsaw, Poland

[3] Department of Microwave and Antenna Engineering, Faculty of Electronics, Telecommunications and Informatics, Gdańsk University of Technology, 80-233 Gdańsk, Poland

Supplementary Information

1. Composite transmission matrix

Incidence of the TEM plane wave onto a periodic layer with (pseudo) randomly distributed inclusions involves the appearance of reflected and transmitted waves, including both TEM and higher-order waves (representing interference effects) with the complex perpendicular propagation constant γ_z given by:

$$\gamma_z = \pm(\alpha + j\beta) = \pm\sqrt{\gamma^2 - \gamma_x^2 - \gamma_y^2} = \pm\frac{2\pi j}{a} \times \sqrt{\varepsilon_\mathrm{m}\mu_\mathrm{m}\left(\frac{a}{\lambda_0}\right)^2 - \left(l_x^2 + l_y^2\right)}, \tag{S1}$$

where the components of the propagation constant $\gamma = \frac{2\pi j}{\lambda_0}\sqrt{\varepsilon_\mathrm{m}\mu_\mathrm{m}}$, $\gamma_x = \frac{2\pi j}{a} l_x$, $\gamma_y = \frac{2\pi j}{a} l_y$ are substituted, l_x, l_y are integers, a denotes the side length of the periodic unit cell, λ_0 is the wavelength in the vacuum at a given frequency, and ε_m and μ_m are relative complex permittivity and permeability of the matrix. Note that at a frequency of 5 GHz, we have λ_0 = 60 mm, which for a < 1 mm, $|\varepsilon_\mathrm{m}| < 3$, and μ_m = 1 involves $\left|\varepsilon_\mathrm{m}\mu_\mathrm{m}\left(\frac{a}{\lambda_0}\right)^2\right| < 10^{-3}$. Therefore, the propagation constants of non-TEM waves equal approximately $\gamma_z \approx \mp\frac{2\pi}{a} \times \sqrt{l_x^2 + l_y^2}$, indicating that they are highly evanescent. Additionally, the more inclusions inside the unit cell, the less significant periodicity of the structure and the impact of interference effects.

In the above study, performed in the gigahertz regime, it is legitimate to assume that the power is transmitted through the composite mainly by TEM waves, and the impact of the higher-order waves is negligible. Thus, a reliable description of the microwave transmission can be performed in terms of transmission matrices $\hat{\boldsymbol{T}}$, which define the relation between electric and magnetic fields at two sides of the layer (denoted by "1" and "2"):

$$\begin{bmatrix} E_2 \\ H_2 \end{bmatrix} = \begin{bmatrix} T_{11} & T_{12} \\ T_{21} & T_{22} \end{bmatrix} \cdot \begin{bmatrix} E_1 \\ H_1 \end{bmatrix}, \tag{S2}$$

where we assume, without loss of generality, the existence of only one polarization. On the other hand, S-parameters accessible in simulations are, in the case of TEM waves, defined as follows:

$$\begin{bmatrix} E_1^- \\ E_2^+ \end{bmatrix} = \begin{bmatrix} S_{11} & S_{12} \\ S_{21} & S_{22} \end{bmatrix} \cdot \begin{bmatrix} E_1^+ \\ E_2^- \end{bmatrix}, \tag{S3}$$

where electric fields corresponding to microwaves propagating in opposite directions (positive “+” and negative “−“) are considered on each side of the layer. The matrix equation (S3) can be transformed into a more convenient form:

$$\begin{bmatrix} E_2^+ \\ E_2^- \end{bmatrix} = \frac{1}{S_{12}} \begin{bmatrix} S_{21}S_{12} - S_{11}S_{22} & S_{22} \\ -S_{11} & 1 \end{bmatrix} \cdot \begin{bmatrix} E_1^+ \\ E_1^- \end{bmatrix}, \tag{S4}$$

with the two sides of the layer considered separately. Furthermore, the following equation can be written to express total E_n and H_n field in terms of E_n^+ and E_n^-:

$$\begin{bmatrix} E_n \\ H_n \end{bmatrix} = \begin{bmatrix} 1 & 1 \\ 1/Z & -1/Z \end{bmatrix} \cdot \begin{bmatrix} E_n^+ \\ E_n^- \end{bmatrix}, \tag{S5}$$

where $Z = \sqrt{\frac{\mu_0 \mu_\mathrm{m}}{\varepsilon_0 \varepsilon_\mathrm{m}}}$ (including absolute vacuum parameters) denotes the characteristic impedance on a layer boundary (equal to the impedance of the matrix at both sides). Finally, the transmission matrix $\widehat{\boldsymbol{T}}$ defined in (S2) can be expressed as:

$$\widehat{\boldsymbol{T}} = \begin{bmatrix} 1 & 1 \\ 1/Z & -1/Z \end{bmatrix} \cdot \frac{1}{S_{12}} \begin{bmatrix} S_{21}S_{12} - S_{11}S_{22} & S_{22} \\ -S_{11} & 1 \end{bmatrix} \cdot \begin{bmatrix} 1 & 1 \\ 1/Z & -1/Z \end{bmatrix}^{-1}. \tag{S6}$$

The matrix $\widehat{\boldsymbol{T}}$ can be further transformed and used to study the effective propagation properties of the composite.

2. Simulation convergence test

To ensure the convergence of the simulation results concerning the thickness of the supercell (a distance between two opposite ports), we performed appropriate tests. We studied vertical stacks of n cubic unit cells with the side length a_1 corresponding to *wt* = 60% (equal to approximately 72 μm), each containing one spherical ceramic inclusion of diameter *d* = 56 μm – the resulting supercells had dimensions $a_1 \times a_1 \times na_1$. As presented in Fig. S1, the permittivity asymptotically converges with the number of layers *n*. The error of the real permittivity is of the order of 0.01 for *n* = 4 layers. The discrepancies between results obtained for supercells with different thicknesses can be assigned to not total extinction of evanescent higher-order microwave modes. The attenuation of a higher-order mode propagating between two opposite ports is proportional to $|e^{-\gamma_z \cdot n a_1}| \approx e^{-n \cdot 2\pi\sqrt{l_x^2 + l_y^2}}$, where l_x, l_y are integers and $\sqrt{l_x^2 + l_y^2} \geq 1$ for higher-order modes (see the section 1). Note that the attenuation does not depend on the cube side length a_1 and only the number of layers n is essential. In the case of fully cubic supercells with N randomly located inclusions (i.e., with dimensions $a \times a \times a$, where $a^3 = Na_1^3$), the attenuation of the higher-order modes is smaller (the corresponding number of layers n equals 1) but interference effects are much less significant due to disordered distribution of the inclusions. For $a = 4a_1$, the corresponding number of inclusions equals 4 × 4 × 4 = 64. Therefore, we concluded that the number of inclusions *N* = 100 provides the proper convergence of the results for any considered value of *wt*.

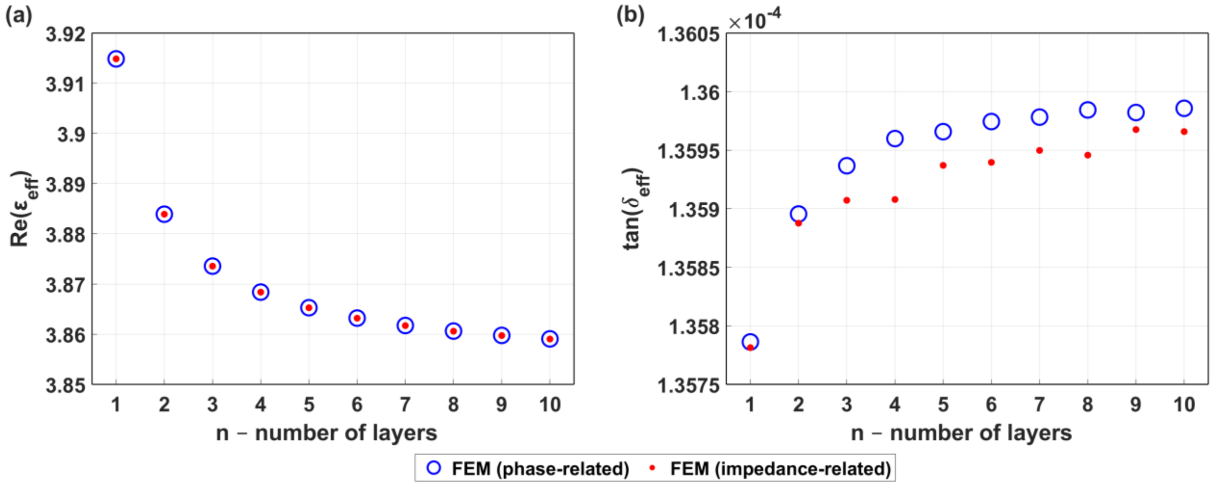

Supplementary Figure S1. Convergence of the simulated dielectric function for ceramic/polypropylene composite (*wt* = 60%) on the example of the structure with a periodic arrangement of the grains (*d* = 56 µm) concerning different thicknesses of the supercell (the number of repeated unit cells).

3. Effect of grain random distribution, size, and shape

3.1. Ceramics/polypropylene composite

The simulations of ceramics/polypropylene composites' real permittivity and loss tangent were performed using periodic sample structures with 100 randomly distributed inclusions with different shapes (balls, ball clusters, cubes, and tetrahedrons). For each inclusion shape, we considered five random geometries and evaluated the dielectric properties for two perpendicular polarization axes. The calculated dielectric properties corresponding to phase-related and impedance-related behaviors (averaged over "+" and "–" propagation directions – see eq. (4) in the main paper) are presented in Fig. S2. We observe statistical fluctuation of the results, however, below the differences caused by different grain shapes. It is expected that for a greater number of inclusions in the model, the fluctuations would be further reduced. Therefore, statistical averaging over multiple random structures and all polarization directions leads to the correct dielectric properties of the desired isotropic composite.

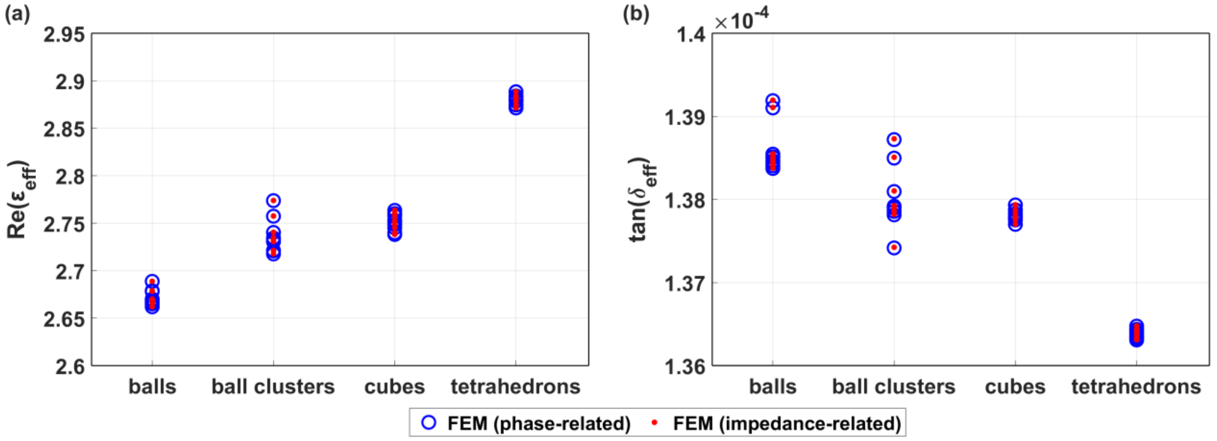

Supplementary Figure S2. Simulated impact of the shape and clustering of the inclusions (*d* = 50 µm) on (a) real part and (b) loss tangent of the ceramics/polypropylene composite's dielectric constant with *wt* = 29.6% at the frequency of 5 GHz. Ten sample results (corresponding to random grain distributions and two perpendicular polarization directions) are presented for each form of the inclusions.

3.2. TiO_2/polypropylene composite

Here, we present supplementary computational studies of grain size, shape, and aggregation effects on the dielectric properties of TiO_2/polypropylene composites. Concerning the discussion of the permittivity of TiO_2 nanograins in the main paper, we performed the simulation using the inclusions' dielectric parameters $\varepsilon'_{\mathrm{TiO2}}$ = 20 and tan δ_{TiO2} = 0.0218. These values are only an estimation but provide a general relation of the composite's dielectric properties with its geometrical structure.

In Fig. S3, we demonstrate using simulations that, similarly to the ceramics/polypropylene composite (see Fig. 3 in the main paper), the grain size has no noticeable effect on the composite's dielectric properties. We studied composites with spherical inclusions – for each grain diameter, we considered a set of five samples with identical random distributions (the geometrical dimensions of entire structures were scaled) and evaluated the dielectric properties for two perpendicular polarization axes. However, convergence issues appear for very small grain and unit cell dimensions in the model, leading to significantly longer computational times and less exact simulation results (due to too small phase differences of the electromagnetic field and too large discretization errors).

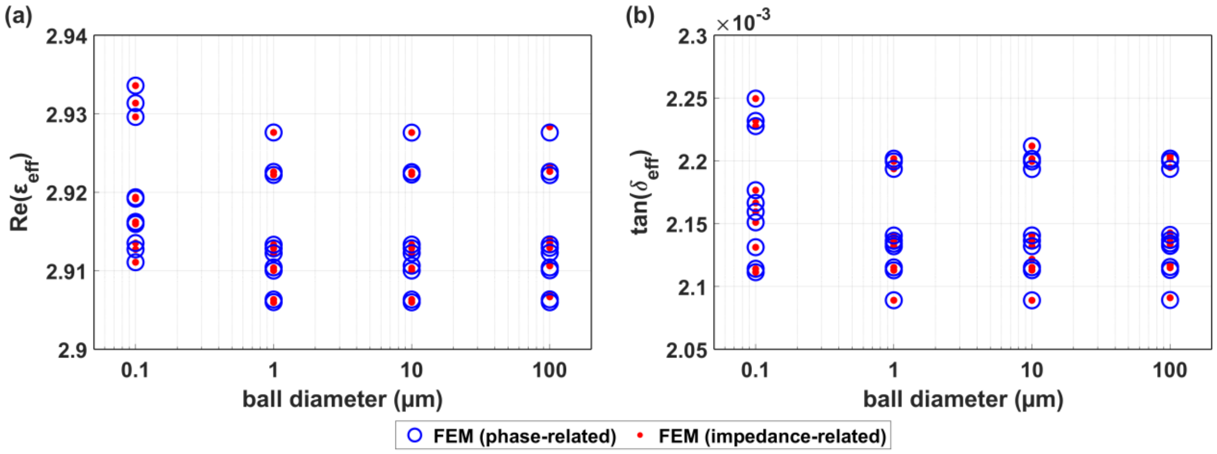

Supplementary Figure S3. Simulated correlation of the size of the spherical inclusions on (a) real part and (b) loss tangent of the TiO_2/polypropylene composite's dielectric constant with *wt* = 40% at the frequency of 5 GHz. Ten sample results (corresponding to random grain distributions and two perpendicular polarization directions) are presented for each size of the inclusions.

In Fig. S4, we study the impact of grain shapes and clustering on the dielectric properties of TiO_2/polypropylene composite. Similar to the studies for ceramics/composite samples, we also consider the statistical fluctuations for inclusions in the form of balls, ball clusters, cubes, and tetrahedrons insignificant compared to the effect of grain shapes. Additionally, we consider a composite with ellipsoid-shaped inclusions with different aspect ratios (using the analytical formula by Bohren and Battan, see eq. (7) in the main paper) and compare them to the Bergman-Milton bounds (green area). Interestingly, the shape of the grains has a noticeable impact on the loss tangent of the composite with lossy inclusions (such as TiO_2) – loss tangent is the smallest for the spherical, non-clustered inclusions.

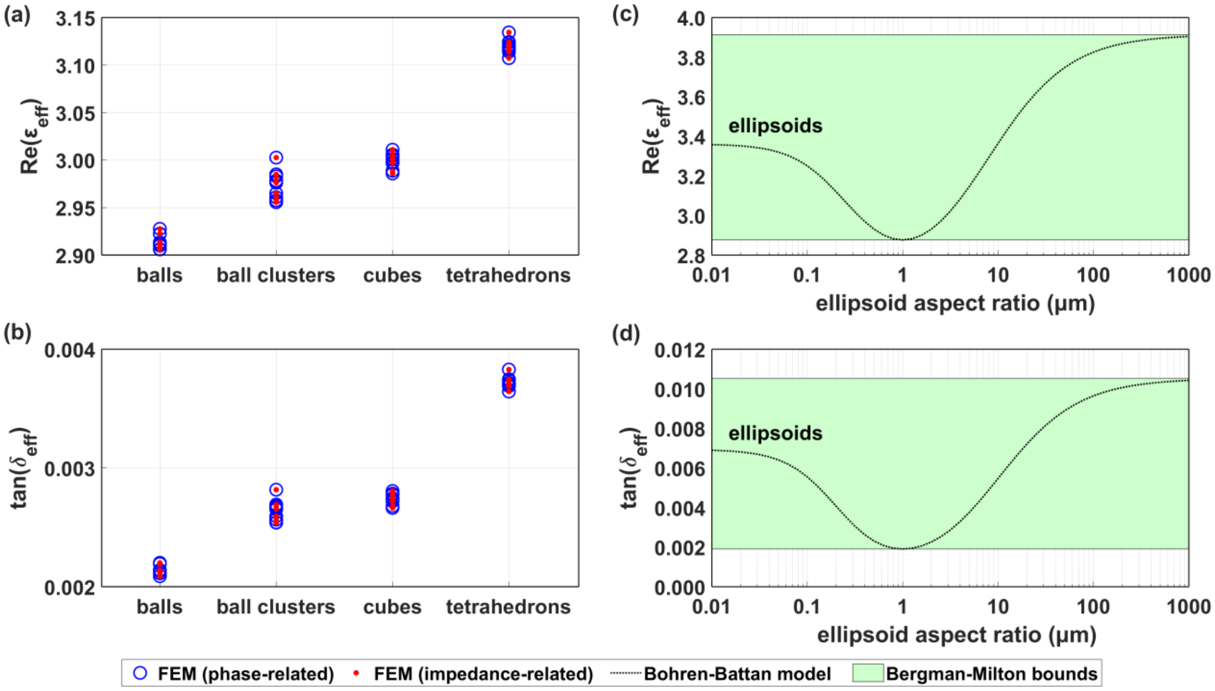

Supplementary Figure S4. Simulated impact of the shape and clustering of the inclusions (d = 1 μm) on (a), (c) real part, and (b), (d) loss tangent of the TiO_2/polypropylene composite's dielectric constant with wt = 40% at the frequency of 5 GHz. Results for non-ellipsoidal (a)-(b) and ellipsoidal (c)-(d) inclusion shapes are presented. In subfigures (a)-(b), ten sample results (corresponding to random grain distributions and two perpendicular polarization directions) are presented for each form of the inclusions. Theoretical Bergman-Milton bounds are presented for comparison in subfigures (c)-(d).
